# Prediction of revision adenoidectomy in pediatric patients: a 10-year retrospective study

**DOI:** 10.3389/fped.2026.1926995

**Published:** 2026-09-15

**Authors:** Yu-Ting Ge, Shi-Yao Yin, Xing-He Zhao, Yue Xu, Hui-E. Zhu

**Affiliations:** 1 Department of Otolaryngology, Children’s Hospital of Soochow University, Suzhou, Jiangsu, China; 2 Department of Ophthalmology, The Fourth Affiliated Hospital of Soochow University, Suzhou, Jiangsu, China

**Keywords:** adenoid hypertrophy, adenoidectomy, nomogram, prediction model, revision surgery

## Abstract

**Objective:**

This study aimed to identify risk factors for revision adenoidectomy in children and to develop and validate a clinical model for individualized risk prediction to support clinical decision-making.

**Methods:**

A total of 222 patients were selected via a nested case-control design from a source cohort of 16,457 children who underwent adenoidectomy at Children's Hospital of Soochow University. The dataset was randomly split into a training set and a validation set at a 7:3 ratio. Independent predictors were identified through univariate and multivariate logistic regression analyses, and seven predictive models were constructed for comparison. The final model's performance was evaluated based on its discrimination (area under the receiver operating characteristic curve, AUC), calibration (calibration plot), and clinical utility (decision curve analysis, DCA).

**Results:**

Multivariate analysis identified six independent risk factors for revision adenoidectomy: adenoid size, surgical instrument type, concurrent tonsillotomy during the initial surgery, allergic rhinitis, otitis media with effusion, and perioperative antibiotic use. Among seven compared models, the logistic regression model showed the best discriminative ability with an AUC of 0.921 in the validation set. A nomogram and a freely accessible web application were developed to facilitate bedside risk calculation.

**Conclusion:**

This study provides the first validated clinical prediction model for revision adenoidectomy in children. The model demonstrates excellent performance and can help clinicians accurately identify children at high risk of needing a revision surgery.

## Introduction

Adenoid hypertrophy is a common condition in children that can cause obstructive sleep apnea, recurrent otitis media with effusion (OME), and chronic sinusitis, making adenoidectomy one of the most frequently performed pediatric surgical procedures (1–4). Despite the widespread use of this operation, a subset of children experienced adenoid regrowth and may require revision surgery. Reported revision adenoidectomy rates vary considerably across studies, ranging from 1.5% to 2.6% in most cohorts (5–8), although some reports have documented rates as high as 9% (9). While this relatively low rate may appear modest, the large volume of primary adenoidectomies performed annually means that a substantial number of children are affected by revision procedures.

Several factors have been identified as potential contributors to adenoid regrowth and revision surgery, including younger age at initial surgery, larger preoperative adenoid size, surgical technique, obesity, and comorbid allergic diseases (7–16). However, these associations have been derived primarily from single-factor analyses or adjusted regression models that identify risk factors but do not provide individualized risk estimation. To date, no validated clinical prediction tool exists that integrates multiple risk factors to estimate a child's individual probability of requiring revision adenoidectomy. Consequently, clinicians lack a systematic approach to stratify postoperative surveillance intensity or to counsel families regarding the likelihood of revision surgery.

To address this gap, we conducted a nested case-control study derived from a 10-year cohort of over 16,000 children who underwent adenoidectomy at a single tertiary pediatric center. We aimed to identify independent risk factors for revision adenoidectomy and to develop and validate a clinical prediction model that could be translated into a practical nomogram and web-based tool for individualized risk assessment. To our knowledge, this represents the first prediction model specifically designed for revision adenoidectomy in children.

## Methods

### Ethics statement

This study was conducted in accordance with the Declaration of Helsinki and was approved by the Institutional Review Board of Children's Hospital of Soochow University (2025CS370).

### Sample size

Contemporary sample size calculation methods for clinical prediction models assume moderate model strength (expected C-statistic < 0.8) (17). Given that our study anticipated a high-performance model based on the strong clinical relevance of candidate predictors, these formula-based approaches are not directly applicable. Pavlou et al. demonstrated that for models with expected high performance (C-statistic > 0.9), the required sample size must be substantially larger than that estimated by Riley's method (18). Consequently, we relied on the well-established Events Per Variable (EPV) criterion to justify sample size adequacy. According to the EPV guideline, each candidate predictor parameter should be supported by at least 10 outcome events to ensure stable model estimation (19, 20).

### Study design

This retrospective nested case-control study included patients who underwent adenoidectomy at Children's Hospital of Soochow University between August 2015 and July 2025. Patients who underwent revision adenoidectomy were identified by screening the entire cohort for duplicate surgical records. A control group was randomly selected from patients who underwent only a single adenoidectomy using computer-generated random numbers, with a sample size twice that of the revision surgery group. The study population was then divided into a training set and a validation set at a 7:3 ratio.

Inclusion criteria were: (1) patients who underwent adenoidectomy; (2) with or without concurrent tonsillotomy; (3) with or without tonsillectomy; (4) with or without grommet insertion; and (5) with or without endoscopic sinus surgery.

Exclusion criteria were: (1) absence of preoperative lateral cephalometric radiographs for adenoid size measurement; (2) postoperative follow-up duration of less than 2 years; (3) adenoid regrowth or hypertrophy after initial adenoidectomy without subsequent revision surgery; and (4) cases with missing data.

### Data collection

All data were collected from inpatient and outpatient medical record systems and the hospital's imaging database. Inpatient records provided information on patient age, sex, height, weight, surgical instruments used, concurrent procedures, revision surgery indications, and perioperative antibiotic use. Outpatient records were reviewed to determine whether relevant comorbidities had been diagnosed, with diagnoses identified based on the ICD-10 disease classification coding system. Specifically, obstructive sleep apnea was coded as G47.301. All imaging data were obtained from the hospital's imaging database. The adenoid-to-nasopharyngeal (A/N) ratio was calculated from preoperative lateral cephalometric radiographs, and imaging evidence of chronic sinusitis and OME was evaluated.

Candidate predictors included patient age, sex, body mass index (BMI), adenoid size (A/N ratio), surgical instruments used, and concurrent procedures (grommet insertion, tonsillotomy, tonsillectomy, endoscopic sinus surgery). Comorbidities considered were OME, allergic rhinitis (AR), chronic sinusitis, and asthma. Perioperative antibiotic administration and postoperative use of recombinant human growth hormone (rhGH) or gonadotropin-releasing hormone analog (GnRHa) were also recorded.

### Outcome

The outcome event was defined as the identification of recurrent adenoid hypertrophy after initial adenoidectomy that met surgical indications and subsequently required revision adenoidectomy.

### Statistical analysis

Statistical analyses were performed using R software (version 4.4.2; R Foundation for Statistical Computing, Vienna, Austria). Continuous variables are presented as mean ± standard deviation (SD) or median with interquartile range (IQR), and group comparisons were conducted using Student's t-test or the Wilcoxon test, as appropriate. Categorical variables are presented as counts and percentages, and group comparisons were performed using the chi-square test or Fisher's exact test. Results are expressed as odds ratios (OR) with 95% confidence intervals (CI). A two-tailed *p*-value < 0.05 was considered statistically significant.

### Model development and comparison

A prediction model for revision adenoidectomy was developed using both univariate and multivariate logistic regression analyses, along with six machine learning algorithms: decision tree (DT), random forest (RF), XGBoost, support vector machine (SVM), k-nearest neighbors (KNN), and naive Bayes model (NBM). Candidate predictors were initially screened using univariate logistic regression, and statistically significant variables were subsequently included in the multivariate logistic regression analysis. Variables retaining statistical significance in the multivariate analysis were used to construct the final logistic regression model.

The confusion matrix parameters were compared across all models, and receiver operating characteristic (ROC) curves were generated. Based on these parameters and the area under the curve (AUC), the model with the strongest predictive performance was selected as the final model.

### Evaluation of model performance

The final model's discriminative ability was assessed using the ROC curve and AUC. Calibration, representing the agreement between predicted and observed probabilities, was evaluated using a calibration plot. Clinical utility, defined as the net benefit of intervening on patients at various threshold probabilities based on model predictions, was examined using decision curve analysis (DCA).

### Nomogram

The final model was presented as a nomogram to predict the risk of revision adenoidectomy. To facilitate clinical application and improve predictive accuracy, the model was deployed as an interactive web application. This application calculates the predicted probability of revision adenoidectomy based on user-input values for each variable.

## Results

### Study population characteristics

Data were derived from 16,457 pediatric patients who underwent adenoidectomy at Children's Hospital of Soochow University between August 2015 and July 2025. Within this cohort, 73 patients underwent a second adenoidectomy, and one patient underwent a third procedure. The mean interval between initial and revision adenoidectomy was 3.6 years. Obstructive sleep apnea was the most common indication for revision surgery (81.08%), followed by OME (14.86%). Tonsillectomy was the most frequently performed concurrent procedure during revision surgery (62.16%), followed by grommet insertion (14.86%). Among the 74 patients who underwent revision adenoidectomy, eight presented with hypertrophy of the torus tubarius, which was also addressed during surgery. Figure 1 presents the study flowchart. Table 1 compares demographic and clinical characteristics between the control group and the revision surgery group, while Table 2 compares the training and validation sets after data partitioning.

**Figure 1:**
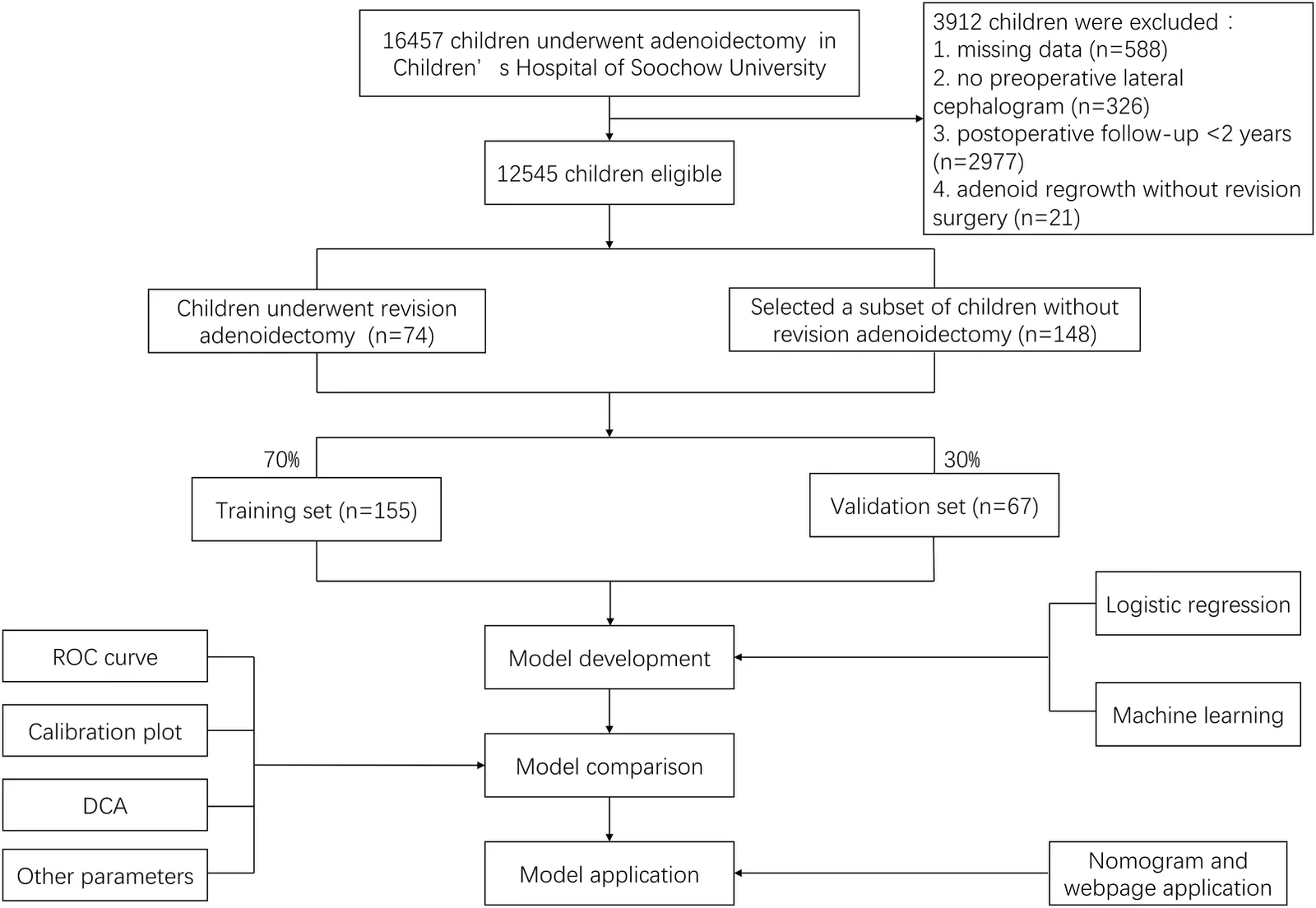
Flow chart of the study design. ROC: receiver operating characteristic; DCA: decision curve analysis.

**Table 1:** Comparisons of demographic and clinical characteristics between control group and revision group.

Variables	Control group (*n* = 148)	Revision group (*n* = 74)	*P*
Age, month	73.00 [59.00–92.25]	50.00 [41.00–59.75]	<0.001
Sex			0.095
Male, *n* (%)	75 (50.68%)	47 (63.51%)	
Female, *n* (%)	73 (49.32%)	27 (36.49%)	
BMI, kg/m^2^	15.64 [14.51–17.51]	15.77 [15.09–17.06]	0.610
A/N (%)	74.00 [65.00–81.25]	89.00 [81.25–100.00]	<0.001
Adenoidectomy instrument			<0.001
Coblation, *n* (%)	88 (59.46%)	8 (10.81%)	
Microdebrider, *n* (%)	60 (40.54%)	66 (89.19%)	
Grommet insertion			0.009
No, *n* (%)	141 (95.27%)	62 (83.78%)	
Yes, *n* (%)	7 (4.73%)	12 (16.22%)	
Tonsillotomy			<0.001
No, *n* (%)	137 (92.57%)	49 (66.22%)	
Yes, *n* (%)	11 (7.43%)	25 (33.78%)	
Tonsillectomy			<0.001
No, *n* (%)	60 (40.54%)	67 (90.54%)	
Yes, *n* (%)	88 (59.46%)	7 (9.46%)	
Endoscopic sinus surgery			0.333
No, *n* (%)	148 (100.00%)	73 (98.65%)	
Yes, *n* (%)	0 (0.00%)	1 (1.35%)	
OME			<0.001
No, *n* (%)	138 (93.24%)	50 (67.57%)	
Yes, *n* (%)	10 (6.76%)	24 (32.43%)	
AR			<0.001
No, *n* (%)	37 (25.00%)	2 (2.70%)	
Yes, *n* (%)	111 (75.00%)	72 (97.30%)	
CRS			0.001
No, *n* (%)	124 (83.78%)	46 (62.16%)	
Yes, *n* (%)	24 (16.22%)	28 (37.84%)	
Asthma			0.024
No, *n* (%)	138 (93.24%)	61 (82.43%)	
Yes, *n* (%)	10 (6.76%)	13 (17.57%)	
Perioperative antibiotics			0.001
No, *n* (%)	45 (30.41%)	41 (55.41%)	
Yes, *n* (%)	103 (69.59%)	33 (44.59%)	
Postoperative rhGH			0.036
No, *n* (%)	148 (100.00%)	71 (95.95%)	
Yes, *n* (%)	0 (0.00%)	3 (4.05%)	
Postoperative GnRHa			0.258
No, *n* (%)	147 (99.32%)	72 (97.30%)	
Yes, *n* (%)	1 (0.68%)	2 (2.70%)	
Revision surgery age, month	NA	92.50 [77.75–109.00]	
Revision surgery indication^a^			
OSA, *n* (%)	NA	60 (81.08%)	
OME, *n* (%)	NA	11 (14.86%)	
TTH, *n* (%)	NA	8 (10.81%)	
Recurrent tonsilitis, *n* (%)	NA	3 (4.05%)	
Concurrent procedure^b^			
Tonsillectomy, *n* (%)	NA	46 (62.16%)	
Grommet insertion, *n* (%)	NA	11 (14.86%)	
Myringoplasty, *n* (%)	NA	1 (1.35%)	

BMI, body mass index; A/N, the adenoid-to-nasopharyngeal ratio; OME, otitis media with effusion; AR, allergic rhinitis; CRS, chronic sinusitis; rhGH, recombinant human growth hormone; GnRHa, gonadotropin-releasing hormone analog; OSA, obstructive sleep apnea; TTH, torus tubarius hypertrophy; NA, not applicable.

a Six patients presented with concurrent OSA and TTH, while two patients had both OME and TTH.

b Three patients underwent concurrent grommet insertion and tonsillectomy.

**Table 2:** Comparisons of demographic and clinical characteristics between training set and validation set.

Variables	Training set (*n* = 155)	Validation set (*n* = 67)	*P*
Age, month	63.00 [51.00–79.50]	68.00 [49.50–81.00]	0.735
Sex			0.279
Male, *n* (%)	81 (52.26%)	41 (61.19%)	
Female, *n* (%)	74 (47.74%)	26 (38.81%)	
BMI, kg/m^2^	15.80 [14.63–17.36]	15.62 [14.43–17.36]	0.660
A/N	0.78 [0.70–0.88]	0.79 [0.72–0.89]	0.626
Adenoidectomy instrument			1.000
Coblation, *n* (%)	67 (43.23%)	29 (43.28%)	
Microdebrider, *n* (%)	88 (56.77%)	38 (56.72%)	
Grommet insertion			0.356
No, *n* (%)	144 (92.90%)	59 (88.06%)	
Yes, *n* (%)	11 (7.10%)	8 (11.94%)	
Tonsillotomy			0.801
No, *n* (%)	131 (84.52%)	55 (82.09%)	
Yes, *n* (%)	24 (15.48%)	12 (17.91%)	
Tonsillectomy			0.349
No, *n* (%)	85 (54.84%)	42 (62.69%)	
Yes, *n* (%)	70 (45.16%)	25 (37.31%)	
Endoscopic sinus surgery			0.302
No, *n* (%)	155 (100.00%)	66 (98.51%)	
Yes, *n* (%)	0 (0.00%)	1 (1.49%)	
OME			0.923
No, *n* (%)	132 (85.16%)	56 (83.58%)	
Yes, *n* (%)	23 (14.84%)	11 (16.42%)	
AR			0.506
No, *n* (%)	25 (16.13%)	14 (20.90%)	
Yes, *n* (%)	130 (83.87%)	53 (79.10%)	
CRS			0.449
No, *n* (%)	116 (74.84%)	54 (80.60%)	
Yes, *n* (%)	39 (25.16%)	13 (19.40%)	
Asthma			0.789
No, *n* (%)	140 (90.32%)	59 (88.06%)	
Yes, *n* (%)	15 (9.68%)	8 (11.94%)	
Perioperative antibiotics			0.445
No, *n* (%)	57 (36.77%)	29 (43.28%)	
Yes, *n* (%)	98 (63.23%)	38 (56.72%)	
Postoperative rhGH			0.555
No, *n* (%)	152 (98.06%)	67 (100.00%)	
Yes, *n* (%)	3 (1.94%)	0 (0.00%)	
Postoperative GnRHa			1.000
No, *n* (%)	153 (98.71%)	66 (98.51%)	
Yes, *n* (%)	2 (1.29%)	1 (1.49%)	

BMI, body mass index; A/N, the adenoid-to-nasopharyngeal ratio; OME, otitis media with effusion; AR, allergic rhinitis; CRS, chronic sinusitis; rhGH, recombinant human growth hormone; GnRHa, gonadotropin-releasing hormone analog.

### Model development and comparison

A total of 16 candidate predictors were screened using univariate logistic regression. Of these, 11 variables demonstrated statistical significance and were subsequently included in the multivariate logistic regression analysis. Six variables were ultimately selected through multivariate logistic regression for model construction, as detailed in Table 3. Seven models were developed, including one logistic regression (LR) model and six machine learning models. Figure 2A summarizes the confusion matrix parameters of these models in the validation set. The LR model performed best in four parameters: sensitivity, negative predictive value, recall, and F1-score. The XGB model performed best in three parameters: specificity, positive predictive value, and precision. The LR model demonstrated superior discriminative ability, with an AUC of 0.921 in the validation set (Figure 2B). Given its highest sensitivity, negative predictive value, F1-score, and overall discriminative performance, the LR model was selected as the final model.

**Table 3:** Results of the univariate and multivariate logistic regression analysis.

Variables	Univariate analysis		Multivariate analysis	
	OR (95% CI)	*P*	OR (95% CI)	*P*
Age	0.946 (0.925–0.967)	0*		
Sex	0.434 (0.215–0.873)	0.019*		
BMI	0.974 (0.856–1.109)	0.69		
A/N	1.131 (1.086–1.178)	0*	1.138 (1.071–1.209)	0*
Adenoidectomy instrument	0.098 (0.039–0.251)	0*	0.176 (0.038–0.816)	0.026*
Grommet insertion	4.11 (1.144–14.773)	0.03*		
Tonsillotomy	5.706 (2.242–14.523)	0*	61.836 (7.994–478.306)	0*
Tonsillectomy	0.087 (0.034–0.223)	0*		
Endoscopic sinus surgery	NA	/		
OME	11.25 (3.867–32.725)	0*	23.432 (4.411–124.470)	0*
AR	14.519 (1.905–110.663)	0.01*	28.315 (1.935–414.281)	0.015*
CRS	2.605 (1.23–5.518)	0.012*		
Asthma	2.667 (0.908–7.828)	0.074		
Perioperative antibiotics	0.341 (0.169–0.685)	0.003*	0.128 (0.030–0.543)	0.005*
Postoperative rhGH	12,86,3198.941 (0-NA)	0.984		
Postoperative GnRHa	2.122 (0.13–34.643)	0.597		

BMI, body mass index; A/N, the adenoid-to-nasopharyngeal ratio; OME, otitis media with effusion; AR, allergic rhinitis; CRS, chronic sinusitis; rhGH, recombinant human growth hormone; GnRHa, gonadotropin-releasing hormone analog; NA, not applicable.

* *P* < 0.05.

**Figure 2:**
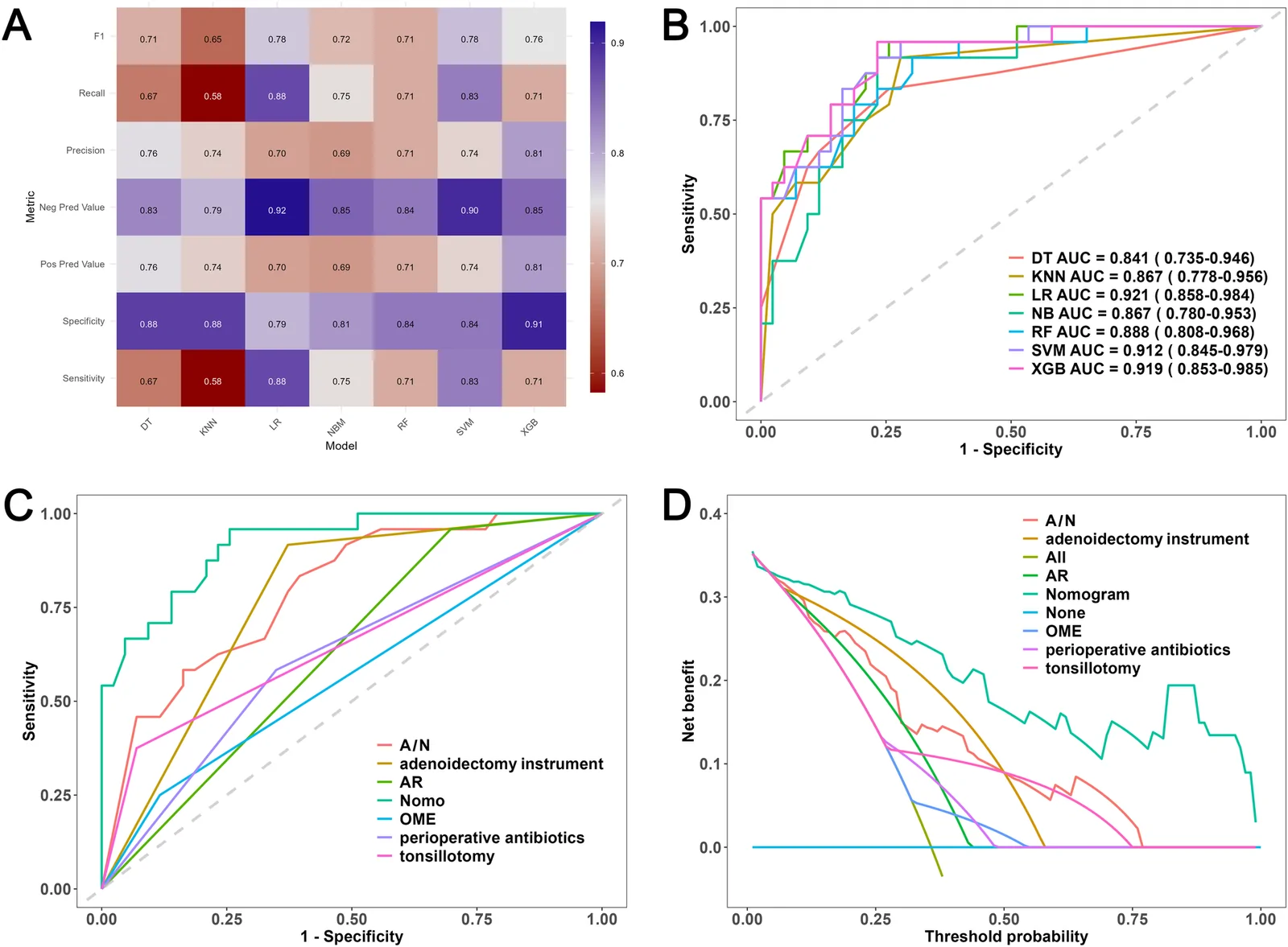
Model comparison and rationality analysis. DT, decision tree; KNN, k-nearest neighbor; LR, logistic regression; NBM, naive Bayes model; RF, random forest; SVM, support vector machine; XGB, XGBoost; A/N, the adenoid-to-nasopharyngeal ratio; AR, allergic rhinitis; OME, otitis media with effusion. **(A)** Heatmaps of the confusion matrix parameters for each model. The LR model performed best in four parameters: sensitivity, negative predictive value, recall, and F1-score. The XGB model performed best in three parameters: specificity, positive predictive value, and precision. **(B)** Combined ROC curves for each model. The LR model achieved the highest AUC (0.921). **(C)** Combined ROC curves for the LR model and each individual variable. **(D)** Combined DCA for the LR model and each individual variable.

### Rationality analysis

A comparison of the ROC curves for the LR model and individual variables (Figure 2C) revealed that the LR model's AUC exceeded that of all single-variable models. DCA for the LR model and individual variables (Figure 2D) demonstrated that the LR model provided a greater net benefit across a wide range of threshold probabilities compared to single-variable modeling strategies.

### Evaluation of model performance

The ROC curve for the LR model in the training and validation sets is shown in Figure 3A. The calibration plot for the validation set is displayed in Figure 3B. With a Brier score of 0.113 and a Hosmer-Lemeshow test *p*-value of 0.746, the model's predicted probabilities closely aligned with observed outcomes. DCA in the validation set is illustrated in Figure 3C. The model provided a higher net benefit than the treat-all or treat-none strategies across threshold probabilities ranging from 3% to 100%, highlighting its potential clinical utility.

**Figure 3:**
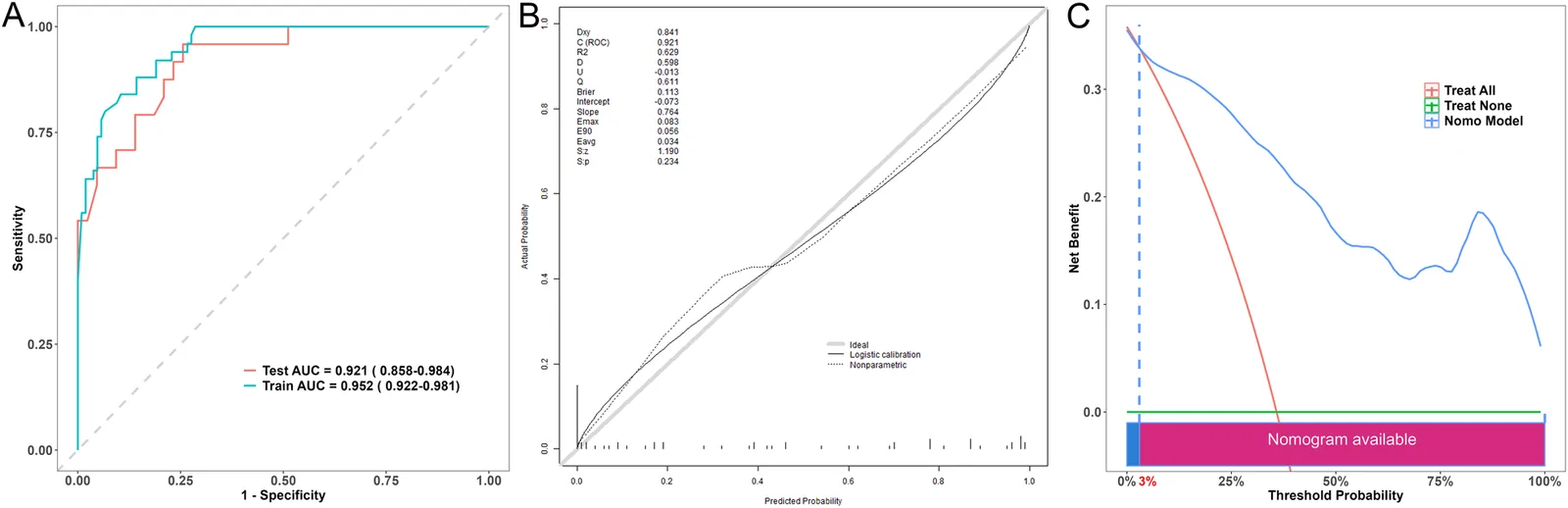
Evaluation of model performance. **(A)** The ROC curve and AUC for the LR model in the training set and the validation set. **(B)** The calibration plot for the LR model in the validation set. **(C)** DCA for the LR model in the validation set.

### Nomogram

Based on the LR model, we constructed a user-friendly nomogram for risk assessment. The value assigned to each variable in the model corresponds to a score on the topmost scale. The scores of the six variables are summed to obtain a total score (331 points), corresponding to a predicted probability of 0.527 (Figure 4A). Additionally, we developed a web-based application. Users can input the values for each variable in the left panel and click the “Predict” button to display the corresponding probability in the right panel. This tool supports multiple sequential calculations and retains a viewable history of these records. The application is available at https://geyuting.shinyapps.io/dynnomapp/ (Figure 4B).

**Figure 4:**
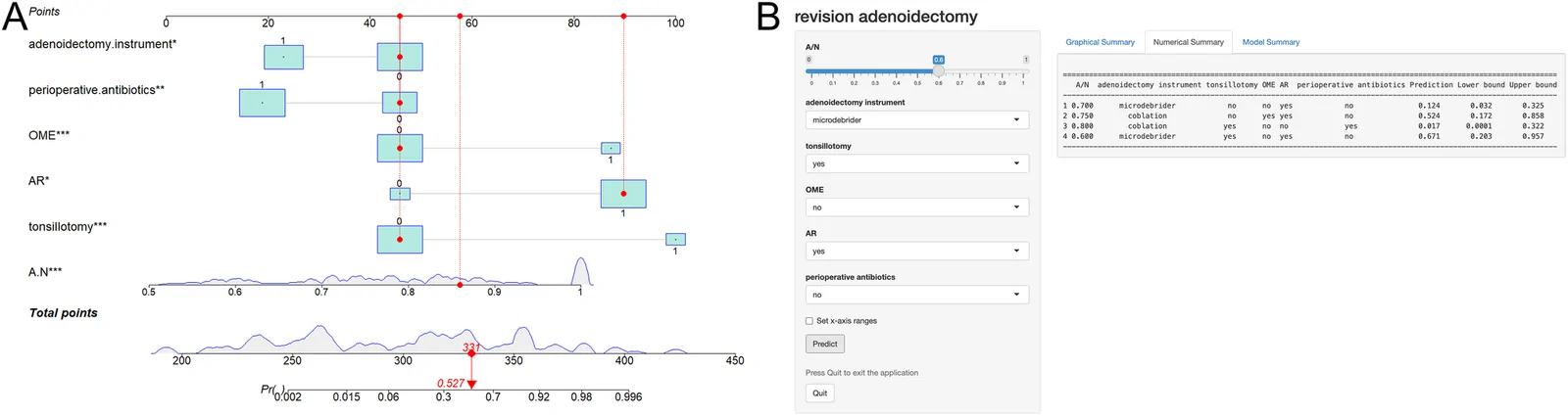
Application of the final model. A/N: the adenoid-to-nasopharyngeal ratio; AR: allergic rhinitis; OME: otitis media with effusion. **(A)** Application of the simplified nomogram. The value assigned to each variable in the model corresponds to a score on the topmost scale. The scores of the six variables are summed to obtain a total score (331 points), corresponding to a predicted probability of 0.527. **(B)** Application of the web application. Enter the value for each variable in the left panel, click the “Predict” button, and the corresponding predicted probability will be displayed in the right panel. The tool supports multiple calculations and review of historical records. The web application is accessible at https://geyuting.shinyapps.io/dynnomapp/.

## Discussion

The revision adenoidectomy rate in this study was 0.6%, which is lower than rates reported elsewhere (5–9, 16). Although some patients developed postoperative recurrence meeting surgical criteria during follow-up, their parents declined revision surgery due to concerns about efficacy and potential further recurrence. We acknowledge that the minimum follow-up period of 2 years may be insufficient to capture all revision adenoidectomy events, particularly given that the mean interval between initial surgery and revision in our cohort was 3.6 years. While extending the minimum follow-up threshold to match the mean interval would have excluded approximately 40% of the total cohort (about 6,700 patients), potentially introducing significant selection bias and compromising sample representativeness, we recognize that some late revisions may have been missed. Loss to follow-up and cases of revision adenoidectomy performed at other institutions were inevitable. However, as our center is a tertiary pediatric and regional referral hospital, the proportion of patients lost to follow-up or seeking care elsewhere is likely low.

Multivariate logistic regression identified the A/N ratio, a measure of adenoid size, as a significant independent risk factor for revision surgery. Larger preoperative adenoid size has been consistently associated with a higher risk of adenoid regrowth and revision adenoidectomy (10, 16, 21). We hypothesize that more active proliferation of pharyngeal lymphoid tissue in these patients predisposes them to postoperative regrowth, a premise requiring further investigation.

Currently, endoscopic microdebrider-assisted adenoidectomy and coblation adenoidectomy are the predominant surgical approaches, both offering excellent surgical exposure, complete resection, and reliable hemostasis (22–24). However, studies have shown that regardless of the surgical technique employed, adenoid residue and regrowth remain possible (25, 26). In our study, we found that compared to coblation, adenoidectomy using a powered microdebrider conferred a 5.7-fold increased risk of revision surgery, consistent with previous reports associating microdebrider use with higher rates of adenoid residue and revision surgery (15, 26). The coblation probe's adjustable angle, combined cutting and coagulation functionality, and better access to recessed areas facilitate more complete resection and reduce adenoid residue. However, the observed association between surgical technique and revision risk may be confounded by unmeasured surgeon-level factors, such as operator experience, technical proficiency, and individual practice patterns. While basic surgeon demographic data were available, they do not fully capture the complex interplay of surgeon-related factors that influence both technique selection and surgical outcomes, and incorporating them into the model would have compromised statistical power. This limitation should be considered when interpreting the findings.

Initial adenoidectomy combined with tonsillotomy was associated with an increased risk of revision surgery in this study. Conversely, Babademez et al. reported that adenoidectomy combined with tonsillotomy resulted in less adenoid regrowth than when combined with tonsillectomy (27). Tonsillotomy is often preferred in younger patients to preserve immunological function. In our cohort, the mean age for concurrent adenoidectomy and tonsillotomy was 3.7 years, with a revision surgery rate of 69.4%, compared to 6.0 years and 26.3% for those not undergoing tonsillotomy. It is important to note that this observed association likely reflects an age effect rather than a direct causal effect of the tonsillotomy procedure itself. Younger patients possess a greater potential for adenoid regrowth and consequently a higher revision surgery risk (7, 8, 10–13). Thus, the association between concurrent tonsillotomy and revision surgery should be interpreted as a statistical association largely driven by age, rather than an independent surgical effect.

Allergic diseases are a known risk factor for adenotonsillar regrowth in children following adenotonsillectomy (28). A meta-analysis indicated that 14% of patients undergoing revision adenoidectomy had AR (5). OME also elevates the risk of revision adenoidectomy (16, 29). In this study, AR and OME were independent risk factors, associated with 28.3-fold and 23.4-fold increased risks of revision surgery, respectively. Among revision surgery cases, 14.9% presented with OME that developed after the initial adenoidectomy, and subsequent examination confirmed adenoid regrowth. Consequently, a high proportion of patients undergoing revision adenoidectomy also received grommet insertion. Bhandari et al. suggested the most common indication for revision adenoidectomy is to improve Eustachian tube dysfunction and avoid repeated grommet insertion (30). Revision adenoidectomy typically occurs 1.3 to 4.3 years after the initial surgery (7, 9, 13, 31, 32), consistent with our mean interval of 3.6 years. The common indications for revision adenoidectomy in this study were obstructive sleep apnea, recurrent OME, and torus tubarius hypertrophy, aligning with previous reports (5, 31–33). Long-term outpatient management is crucial, as many patients with adenoid hypertrophy have comorbidities like AR, OME, and asthma, necessitating close attention to those at high recurrence risk.

We also investigated the relationship between medication use and revision adenoidectomy, finding that perioperative antibiotic administration was associated with a reduced risk of revision surgery in our cohort. Importantly, this association should not be interpreted as evidence of a causal protective effect of antibiotics, for several reasons. First, the analysis did not account for antibiotic class, dose, timing, or duration, as this information was not systematically available. Second, the association may be subject to confounding by indication or treatment-selection bias. Third, our finding is inconsistent with previous studies that reported patients receiving multiple perioperative antibiotic courses are more prone to adenoid regrowth or revision surgery (16, 34). The cohort in this study consisted of Chinese children, a population distinct from those in the cited reports, and differences in prescribing practices may contribute to the discrepancy. Notably, the primary objective of this study was to develop a prediction model for risk stratification rather than to establish causal relationships. In this context, perioperative antibiotic use is included as a predictor because it improved model performance. Therefore, this finding should be considered primarily as a hypothesis-generating observation that requires further validation in future prospective studies designed to test the effects of perioperative antibiotic regimens on adenoid regrowth. Until such studies are conducted, the association should be interpreted with caution.

Although postoperative rhGH use was not included in the final model, we noted that three patients in the revision surgery group had received long-term rhGH injections after their initial adenoidectomy due to growth hormone deficiency or Turner syndrome, whereas no patients in the control group received such treatment. Common adverse effects of growth hormone therapy include adenoid hypertrophy, although a dose-dependent effect remains controversial (35, 36), and some authors have suggested that progressive adenoid hypertrophy during rhGH treatment may represent a physiological process rather than a treatment-specific effect (37).

The increasing application of machine learning in prediction models stems from its ability to capture complex, non-linear relationships. However, these algorithms risk overfitting in smaller datasets and lack interpretability, hindering clinical adoption (38, 39). In this study, logistic regression outperformed machine learning algorithms. This finding reflects two factors: our large-scale foundational cohort provided stable sample distribution, and the core predictors have strong clinical associations with revision surgery. When predictor-outcome relationships are predominantly linear, logistic regression efficiently captures these effects without overfitting noise. Our final model, presented as a nomogram and web application, thus combines high performance with transparency and clinical utility.

The sample size of this study meets the well-established Events Per Variable criterion, with the number of outcome events per predictor exceeding the recommended threshold of 10, which is widely accepted for stable logistic regression model development. The model's excellent performance in the validation set provides direct empirical evidence that the sample size was sufficient. Beyond sample size, this study has several limitations. First, it is a single-center retrospective study lacking an external validation set; therefore, the model's generalizability requires confirmation through multi-center studies with larger sample sizes. Additionally, the use of a single 7:3 hold-out split for internal validation may introduce variability in performance estimates, particularly given the modest sample size; future studies with larger cohorts should consider bootstrap or k-fold cross-validation to further validate the model's performance. Second, the presence of patient comorbidities was determined based on diagnostic codes from the medical record database, and the diagnostic basis for certain patients may be insufficient. Finally, we acknowledge that the outcome of revision adenoidectomy is influenced by parental preference and health-seeking behavior, and we were unable to assess recurrent cases that did not progress to surgical intervention, as systematic postoperative imaging was not performed in asymptomatic patients. Therefore, our model predicts the risk of undergoing revision surgery rather than biological recurrence. This distinction should be considered when interpreting our findings.

## Conclusion

This study identified adenoid size, surgical instrument type, concurrent tonsillotomy during initial adenoidectomy, AR, OME, and perioperative antibiotic use as risk factors for revision adenoidectomy. A clinical prediction model based on these factors demonstrated excellent predictive performance. This risk assessment tool enables clinicians to accurately identify patients at high risk of requiring revision surgery.

## Data Availability

The original contributions presented in the study are included in the article, further inquiries can be directed to the corresponding author.
