# Correction: Prediction of revision adenoidectomy in pediatric patients: a 10-year retrospective study

**DOI:** 10.3389/fped.2026.1999410

**Published:** 2026-10-09

**Authors:** Yu-Ting Ge, Shi-Yao Yin, Xing-He Zhao, Yue Xu, Hui-E. Zhu

**Affiliations:** 1 Department of Otolaryngology, Children’s Hospital of Soochow University, Suzhou, Jiangsu, China; 2 Department of Ophthalmology, The Fourth Affiliated Hospital of Soochow University, Suzhou, Jiangsu, China

**Keywords:** adenoid hypertrophy, adenoidectomy, nomogram, prediction model, revision surgery

There was a mistake in Table 2 as published. The data for the two surgical instrument rows, “Coblation” and “Microdebrider,” were inadvertently interchanged. The data currently shown under “Coblation” should appear under “Microdebrider,” and vice versa. The corrected Table 2 appears below.

**Table 2:** Comparisons of demographic and clinical characteristics between training set and validation set.

Variables	Training set (*n* = 155)	Validation set (*n* = 67)	*P*
Age, month	63.00 [51.00–79.50]	68.00 [49.50–81.00]	0.735
Sex			0.279
Male, *n* (%)	81 (52.26%)	41 (61.19%)	
Female, *n* (%)	74 (47.74%)	26 (38.81%)	
BMI, kg/m^2^	15.80 [14.63–17.36]	15.62 [14.43–17.36]	0.660
A/N	0.78 [0.70–0.88]	0.79 [0.72–0.89]	0.626
Adenoidectomy instrument			1.000
Coblation, *n* (%)	67 (43.23%)	29 (43.28%)	
Microdebrider, *n* (%)	88 (56.77%)	38 (56.72%)	
Grommet insertion			0.356
No, *n* (%)	144 (92.90%)	59 (88.06%)	
Yes, *n* (%)	11 (7.10%)	8 (11.94%)	
Tonsillotomy			0.801
No, *n* (%)	131 (84.52%)	55 (82.09%)	
Yes, *n* (%)	24 (15.48%)	12 (17.91%)	
Tonsillectomy			0.349
No, *n* (%)	85 (54.84%)	42 (62.69%)	
Yes, *n* (%)	70 (45.16%)	25 (37.31%)	
Endoscopic sinus surgery			0.302
No, *n* (%)	155 (100.00%)	66 (98.51%)	
Yes, *n* (%)	0 (0.00%)	1 (1.49%)	
OME			0.923
No, *n* (%)	132 (85.16%)	56 (83.58%)	
Yes, *n* (%)	23 (14.84%)	11 (16.42%)	
AR			0.506
No, *n* (%)	25 (16.13%)	14 (20.90%)	
Yes, *n* (%)	130 (83.87%)	53 (79.10%)	
CRS			0.449
No, *n* (%)	116 (74.84%)	54 (80.60%)	
Yes, *n* (%)	39 (25.16%)	13 (19.40%)	
Asthma			0.789
No, *n* (%)	140 (90.32%)	59 (88.06%)	
Yes, *n* (%)	15 (9.68%)	8 (11.94%)	
Perioperative antibiotics			0.445
No, *n* (%)	57 (36.77%)	29 (43.28%)	
Yes, *n* (%)	98 (63.23%)	38 (56.72%)	
Postoperative rhGH			0.555
No, *n* (%)	152 (98.06%)	67 (100.00%)	
Yes, *n* (%)	3 (1.94%)	0 (0.00%)	
Postoperative GnRHa			1.000
No, *n* (%)	153 (98.71%)	66 (98.51%)	
Yes, *n* (%)	2 (1.29%)	1 (1.49%)	

BMI, body mass index; A/N, the adenoid-to-nasopharyngeal ratio; OME, otitis media with effusion; AR, allergic rhinitis; CRS, chronic sinusitis; rhGH, recombinant human growth hormone; GnRHa, gonadotropin-releasing hormone analog.

The original version of this article has been updated.

